# Quantification of Volatile Acetone Oligomers Using Ion-Mobility Spectrometry

**DOI:** 10.1155/2021/6638036

**Published:** 2021-08-02

**Authors:** Tobias Hüppe, Dominik Lorenz, Felix Maurer, Tobias Fink, Ramona Klumpp, Sascha Kreuer

**Affiliations:** Center of Breath Research, Department of Anaesthesiology, Intensive Care and Pain Therapy, Saarland University Medical Center, Homburg, Saarland 66424, Germany

## Abstract

**Background:**

Volatile acetone is a potential biomarker that is elevated in various disease states. Measuring acetone in exhaled breath is complicated by the fact that the molecule might be present as both monomers and dimers, but in inconsistent ratios. Ignoring the molecular form leads to incorrect measured concentrations. Our first goal was to evaluate the monomer-dimer ratio in ambient air, critically ill patients, and rats. Our second goal was to confirm the accuracy of the combined (monomer and dimer) analysis by comparison to a reference calibration system.

**Methods:**

Volatile acetone intensities from exhaled air of ten intubated, critically ill patients, and ten ventilated Sprague-Dawley rats were recorded using ion-mobility spectrometry. Acetone concentrations in ambient air in an intensive care unit and in a laboratory were determined over 24 hours. The calibration reference was pure acetone vaporized by a gas generator at concentrations from 5 to 45 ppb_v_ (parts per billion by volume).

**Results:**

Acetone concentrations in ambient laboratory air were only slightly greater (5.6 ppb_v_; 95% CI 5.1–6.2) than in ambient air in an intensive care unit (5.1 ppb_v_; 95% CI 4.4–5.5; *p* < 0.001). Exhaled acetone concentrations were only slightly greater in rats (10.3 ppb_v_; 95% CI 9.7–10.9) than in critically ill patients (9.5 ppb_v_; 95% CI 7.9–11.1; *p* < 0.001). Vaporization yielded acetone monomers (1.3–5.3 mV) and dimers (1.4–621 mV). Acetone concentrations (ppb_v_) and corresponding acetone monomer and dimer intensities (mV) revealed a high coefficient of determination (*R*^2^ = 0.96). The calibration curve for acetone concentration (ppb_v_) and total acetone (monomers added to twice the dimers; mV) was described by the exponential growth 3-parameter model, with an *R*^2^ = 0.98.

**Conclusion:**

The ratio of acetone monomer and dimer is inconsistent and varies in ambient air from place-to-place and across individual humans and rats. Monomers and dimers must therefore be considered when quantifying acetone. Combining the two accurately assesses total volatile acetone.

## 1. Introduction

Acetone is one of the most volatile molecules in human breath and responsible for the odor of decaying apple in expired air. Decarboxylation of acetoacetate and oxidation of isopropanol are the two major physiological sources of acetone in humans [1]. Acetone is an important physiological marker of lipolysis starvation [2]. It is elevated in various disease states including diabetes mellitus [3], isopropyl-alcohol intoxication [4], ketogenic and Atkins diets [5], lung tumors [6], and infections [7]. Assessing blood acetone concentration from exhaled gas might be useful for clinicians and patients. For example, diabetics might use expired breath to determine whether they are becoming ketotic.

Measuring acetone in exhaled air is complicated by the fact that the molecule might be present as both monomers and dimers [8], and the relationship is inconsistent. Our first goal was therefore to evaluate the monomer-dimer ratio in ambient air of an intensive care unit and a laboratory, as well as in exhaled breath of critically ill patients and rats.

To the extent that the monomer-dimer ratio varies, accurate assessment of total acetone in expired breath will require determining the concentrations of each species and adding the monomer concentration to twice the dimer concentration. Our second goal was thus to confirm that this combined analysis approach is accurate using a reference calibration system.

Our study addresses two important issues: (1) simple quantification of volatile acetone oligomers in exhaled air using ion-mobility spectrometry and (2) quantification of accuracy by comparison to values from a calibration gas generator. Our results will facilitate future investigations of volatile acetone as a biomarker.

## 2. Materials and Methods

### 2.1. Multicapillary Column Ion-Mobility Spectrometry

Volatile acetone intensities were recorded as described previously using a MCC-IMS (BreathDiscovery, B&S Analytik, Dortmund, Germany) [9, 10]. Preseparation of volatile acetone by multicapillary columns resulted in compound retention time (RT), analysis by ion-mobility spectrometry in drift time (1/K0). Acetone monomers and dimers were identified in the chromatogram by Visual Now 3.6 (B&S Analytik, Dortmund, Germany) and comparison with an existing database (BS-MCC/IMS-analytes database, version 1209, B&S Analytik, Dortmund, Germany) [11] as well as pure substance measurements. Intensity of total volatile acetone was calculated:

Total acetone intensity (V) = acetone monomer intensity (V) + (2 *∗* acetone dimer intensity (V)).

### 2.2. Calibration Gas Generator

Acetone pure substance (99.9%, Sigma Aldrich, Seelze, Germany) was vaporized at a relative humidity of 100% and a temperature of 37°C in steps of 5 ppb_v_ and concentrations ranging from 5 to 45 ppb_v_ using a calibration gas generator (HovaCAL, Inspire Analytical Systems, Oberursel, Germany) as previously described [12]. At each concentration, five 10 ml gas samples were aspirated from the calibration gas generator through a polytetrafluoroethylene tube (Bohlender, Grünsfeld, Germany). Blank measurements were interposed between each concentration step.

### 2.3. Room Air

Acetone concentrations in ambient air from an intensive care unit and from a laboratory were determined using MCC-IMS, each over a period of 24 hours with a sampling interval of 30 minutes.

### 2.4. Patients

With approval from the responsible ethics committee (identification number 232/14, Ärztekammer Saarland, Saarbrücken, Germany) and in accordance with the STROBE guidelines, volatile acetone concentrations were evaluated in ten sedated, intubated, and mechanically ventilated adults from a surgical intensive care unit. Written consent was obtained from either patients or legal guardians.

Patients were ventilated with an intensive care respirator (EVITA 4, Dräger, Lübeck, Germany) with ventilation parameters and oxygen concentrations adjusted to maintain physiological blood gas values. A polytetrafluoroethylene tube was connected near each patient's endotracheal tube. 10 ml mixed inspired and expired gas samples were aspirated from the breathing circuit at 30-minute intervals for a period of twelve hours.

### 2.5. Animals

With authorization by the local agency of animal protection (approval number 41/2014, Ministerium für Umwelt und Verbraucherschutz, Saarbrücken, Germany) and in accordance with the German Animal Welfare Act and ARRIVE guidelines, we studied ten male Sprague-Dawley rats (200–300 g bodyweight, age 8–10 weeks, Charles River, Sulzfeld, Germany). Each was anaesthetized, tracheotomized, and ventilated for a period of 24 hours as described previously [9, 10]. 10 ml expired gas samples were aspirated from the ventilator tubing system through polytetrafluoroethylene tubes every 20 min and analyzed using MCC-IMS [13].

### 2.6. Statistics

We used SigmaPlot (version 12.5, Systat Software, Erkrath, Germany) for statistical analyses. The correlation between voltages and concentrations was calculated using Pearson correlation. Monomer and dimer fraction versus concentration in ppb_v_ was plotted on a 3D-graph. We used *t* or Mann–Whitney rank sum tests to compare ambient acetone concentrations from intensive care unit and laboratory air. To compare patients and rats, we used one-way ANOVA. A two-tailed *p* value < 0.05 was considered statistically significant.

## 3. Results

Acetone concentration in ambient air was slightly higher in laboratory (5.6 ppb_v_; 95% CI 5.1–6.2) than in intensive care unit (5.1 ppb_v_; 95% CI 4.4–5.5; *p* < 0.001). Intensities of monomers and dimers as well as dimer fraction and concentrations of volatile acetone in ambient air are presented in Table 1.

Exhaled acetone concentration was slightly higher in rats (10.3 ppb_v_; 95% CI 9.7–10.9) than in critically ill patients (9.5 ppb_v_; 95% CI 7.9–11.1; *p* < 0.001). Seven patients had concentrations between 4 and 6 ppb_v_, whereas three patients displayed substantially greater concentrations in exhaled air (8.3, 12.9, and 48.3 ppb_v_). Dimer fraction was comparable in the rats (95%, 95% CI 95-96) and critically ill patients (90%, 95% CI 89–91) (Table 1).

**Figure 1:**
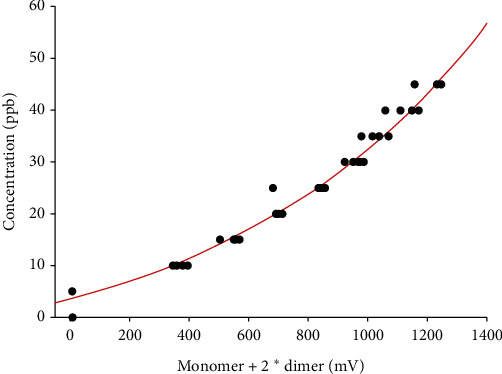
Calibration curve of acetone concentration (ppb_v_) vs. acetone monomer and 2^*∗*^ acetone dimer (mV); *R*^2^ = 0.98. Acetone concentration (ppb_v_) = -10.3 + 13.8*∗*exp (1.1*∗*total acetone intensity (V)).

Acetone vaporization resulted in 45 data triplets containing concentration (ppb_v_), monomer intensity (volts), and dimer intensity (volts). Monomers yielded intensities from 1.3 to 5.3 mV and dimers intensities from 1.4 to 621 mV. Calibration curve of acetone concentration (ppb_v_) vs. total acetone intensity (mV) fits an exponential growth 3-parameter model with an *R*^2^ = 0.98 (Figure 1).

**Figure 2:**
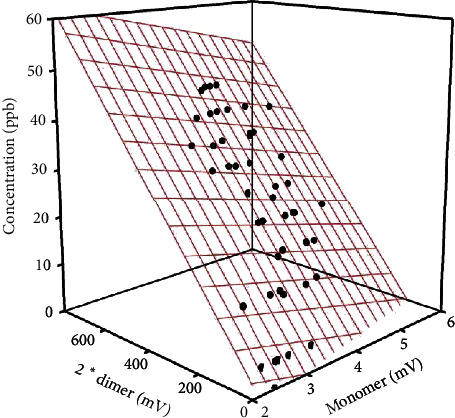
Acetone concentrations (ppb_v_) and corresponding acetone monomer and dimer intensities (mV) measured by MCC-IMS; *R*^2^ = 0.96. Acetone concentration (ppb_v_) = 8.41–2928*∗*acetone monomer intensity (mV) + 74*∗*acetone dimer intensity (mV).

Correlation analysis of calibration of monomer (mV) versus dimer (mV) versus concentration (ppb_v_) revealed the coefficient of determination of *R*^2^ = 0.96 (Figure 2).

## 4. Discussion

The monomer-dimer ratios varied slightly across locations and in the exhaled breath of critically ill patients and rats. However, we detected comparable concentrations of volatile acetone in laboratory and intensive care unit. Dimer fraction was slightly increased in the intensive care ward without reaching a statistical significance. Thus, concentrations and the distribution pattern of volatile acetone oligomer vary, but only to a small extent. Although our results show significant differences with narrow confidence intervals between different environments, these findings are specific for our building and might be clinical negligible. They merely reflect a snapshot of specific daytime with individual staff and equipment and might differ at another point in time limiting our data. There are multiple plausible sources of acetone emission in laboratories including chemical products, cleaning and disinfection solutions, pharmaceuticals, antiseptics, and the human exhalome [14]. However, almost identical dimer fractions in ambient air might be the result of comparable humidity and temperature in laboratory and intensive care unit.

Acetone concentration in ambient air can be rapidly determined using MCC-IMS but might be dependent on numerous influencing factors. Confirming our results, Bessonneau et al. reported widely variable acetone concentrations between different sampling sites in hospital using gas chromatography-mass spectrometry. In addition, they observed a significant temporal variability in concentrations levels of acetone due to multiples sources of emissions [14]. Compared to public building and private houses, hospital showed higher acetone concentrations in ambient air [15].

We demonstrated substantial higher expired acetone concentrations in rats compared to ventilated critically ill patients. Dimer fractions were similar in both groups but almost twice as high as in ambient air. Our data indicate narrow confidence intervals for the most standardized model in rats, receiving a continuous glucose solution and slightly greater interindividual variability in ventilated patients suggesting numerous influencing factors during critical illness. Nevertheless, critically ill patients are nourished artificial. Continuous supply via the gastric tube provides sufficient energy intake preventing marked ketogenesis and acetone production [16]. However, interindividual variability in exhaled acetone might be caused by postaggression syndrome, increased lipolysis, and accumulation of fatty acids and ketones during critical illness. We were able to show that volatile acetone concentrations differ in expired air of various subjects. Additionally, MCC-IMS enables sufficient quantification of acetone even in humid expired air.

There is widespread evidence for the correlation between blood glucose levels and exhaled acetone concentrations in critically ill patients [17]. Acetone derives from acetoacetate and degradation of ketone bodies explaining these findings [18]. However, large variation in expired acetone levels in critically ill patients has been described before [19–21], limiting the accuracy of acetone as a marker for blood glucose prediction in critical care medicine. Furthermore, coexisting diabetes mellitus [3] and infections [7] during critical illness influence the acetone metabolism and result in unpredictable expired acetone concentrations. This might explain findings of Scholpp and colleagues, detecting comparable exhaled acetone concentrations between healthy volunteers and critically ill, mechanically ventilated patients [22]. In contrast to humans, we demonstrated in a previous study, a poor correlation between volatile acetone and blood glucose levels in rats but a trend toward lower acetone concentrations with the cumulative input of glucose [23]. However, differences in exhaled acetone concentrations between critically ill patients and rats are speculative and might depend on several influencing factors.

Different subjects showed various distribution patterns of acetone monomer and dimer. Variations were significant between groups with relatively narrow confidence intervals in the highly standardized rat model but greater spread among the inhomogeneous group of patients. Usually, breathing tubes of ventilated patients were connected to a heat-and-moisture exchanging filter. This results in increased humidity and higher temperature of inspired air. Thus, higher fraction of acetone monomer is the consequence explaining our findings. Compared to this, rats were ventilated without breathing filters. Therefore, lower temperature and less humidity of inhaled air are responsible for higher dimer fraction. Furthermore, it can be expected that oxygen and nitrogen content, respectively, might influence acetone monomer and dimer formation as well; the higher humidity of oxygen compared to nitrogen accelerates the formation of acetone water clusters, so less dimer is observed using higher oxygen fractions. In our study, we ventilated rats with 21% oxygen. Usually, critically ill patients need higher oxygen fractions explaining our findings. It is apparent from our results that the condition of inspired air contributes to a considerable extent to the distribution pattern of monomer and dimer in expired air. Consequently, monomer formation might have a significant impact in breath gas analysis of ventilated critically ill patients.

Acetone monomer and dimer have a significant impact on total acetone concentration. We demonstrated a substantial correlation between monomer, dimer, and acetone concentrations exhibiting a high coefficient of determination. Considering total acetone intensity consisting of monomer and double dimer intensity correlation yielded even superior dependency. Ratio of monomer to dimer formation depends on several influencing factors: high concentration [24, 25], low humidity [26], and low temperature [27] result in increased dimer formation. Formation of protonated dimer could be observed for several volatile compounds, depending on further physicochemical properties that are not completely understood and predictable [26]. Acetone dimer can be expected to be a proton bound, two-fold ionic complex [28]. However, the protonation leads to higher carbonyl activity side reactions such as aldol addition and even aldol condensation can occur. Nevertheless, it has been proven (with ab initio calculations and semiempirically) that the most likely and most stable isomer is the proton bound dimer [29]. Acetone is known to have a higher proton affinity (812 kJ/mol) compared to water (697 kJ/mol). Therefore, acetone can be detected with great sensitivity using the atmospheric pressure chemical ionization process in MCC-IMS [30].

Although higher protonated oligomers of acetone are theoretically possible, we did not observe higher oligomers than the dimer. Presumably, the acetone dimer water clusters are stable at given conditions and atmospheric pressure. Due to short time being ionized, not even the next higher oligomer, the acetone trimer, can be formed in a measurable amount for our given method. It is apparent from our results that MCC-IMS is suitable for bedside and near real-time quantification of volatile acetone even in exhaled humid breath on condition that monomer is not neglected. Real-time measurement and low-instrumental requirement (atmospheric pressure) of MCC-IMS is a huge advantage against the gold standard method gas chromatography-mass spectrometry for quantification of acetone in human breath. Latter needs a vacuum where molecules get ionized. At this, protonated acetone molecules have less time to form dimers and undergo side reactions caused by short lifespan after ionization in vacuum. Signals of dimer and potential isomers are much smaller using gas chromatography-mass spectrometry. Furthermore, since proton bound dimer and aldol adduct have the same *m*/*z* = 117 and the same retention time, it is challenging to separate those two species using gas chromatography-mass spectrometry, since they can only be formed after ionization and therefore have same retention times.

We selected MCC-IMS for quantification of volatile acetone and analysis of expired air in both, humans and rats. Advantages compared to other technologies include bedside online-measurements with near real-time data acquisition, low technical and financial expenditure, and high sensitivity with detection limit down to the ng L^−1^ and pg L^−1^ range [31]. Direct investigation of humid exhaled air, minimal weight, size, and power consumption, and no required vacuum are major benefits of this technology over gas chromatography-mass spectrometry. This approach enables serial bedside measurements even on the intensive care ward and during long-term measurements in ventilated rats [10]. MCC-IMS provides intensities (millivolt) as a surrogate of quantity rather than concentrations, but calibration reveals an excellent correlation between monomer, dimer, and acetone concentration with a high coefficient of determination.

The acetone concentrations we observed in ambient and expired air are specific for our setting and will presumably differ at least somewhat in other settings. It can be assumed that especially critically ill patients exhibit numerous influencing and contributing factors in quantification of volatile acetone. Thus, comparing concentrations of expired acetone between different subjects must be performed with caution. Second, we conducted gas sampling in mixed, inspired, and expired air of the tubing system of ventilated humans and rats. Alveolar sampling during expiration might result in considerable higher intensities, higher concentrations, and more reliable results. Therefore, comparing our results to other studies might be challenging. Indeed, other authors reported higher concentrations of expired acetone [19–21]. Third, outlined calibration curves and correlations between acetone monomer and dimer are specific for our individual device and not transferable in general. Other devices require individual calibration. Different circumstances with divergent humidity, temperature, and gas composition might reveal different findings. And finally, it remains unclear to what extent dimers are formed during ionization in the IMS device and to what extent endogenous dimers contribute to this. Nevertheless, we were able to show that oligomers are sufficiently quantifiable using IMS regardless of their origin.

## 5. Conclusions

In summary, acetone may be a clinically meaningful biomarker. However, the ratio of acetone monomer and dimer is inconsistent and varies (slightly) in ambient air from place-to-place and across individual humans and rats. Monomers and dimers must therefore be considered when quantifying volatile acetone. Ion-mobility spectrometry combines monomers and dimers in the analysis, and precise measurements require that each be quantified.

## Figures and Tables

**Table 1:** Monomer, dimer, and total acetone intensity (mV).

	Intensity (mV)			Dimer (%)	Concentration (ppb_v_)
	Monomer	Dimer	Total		
Intensive care unit	40 (37–46)	22 (18–27)	84 (70–97)	48 (46–50)	5.1 (4.4–5.5)
Laboratory	74 (52–96)	21 (17–25)	116 (88–144)	44 (39–48)	5.6 (5.1–6.2)^#^
Critically ill patients	15 (12–18)^*∗*^	106 (85–126)^*∗*^	226 (185–268)^*∗*^	90 (89–91)	9.5 (7.9–11.1)^*∗*^
Rats	6 (6-6)^*∗*^	127 (118–135)^*∗*^	259 (241–276)^*∗*^	95 (95-96)	10.3 (9.7–10.9)^*∗*^

Monomer, dimer, and total acetone intensity ((mV); total acetone intensity (mV) = acetone monomer intensity (mV) + (2^*∗*^ acetone dimer intensity (mV))), dimer peak fraction of total acetone peak intensity, and concentrations (ppb_v_) in ambient air of intensive care unit, laboratory, critically ill patients, and rats. Data are expressed as means (95% CI); ^#^*p*<0.001 significant higher concentration of volatile acetone in laboratory vs. intensive care unit (Mann–Whitney rank sum test); ^*∗*^*p* < 0.001 significant different intensities and concentrations of volatile acetone in critically ill patients vs. rats (one-way ANOVA).

## Data Availability

The data used to support the findings of this study are available from the corresponding author upon request.
